# Effects of food properties on chewing in pigs: Flexibility and stereotypy of jaw movements in a mammalian omnivore

**DOI:** 10.1371/journal.pone.0228619

**Published:** 2020-02-07

**Authors:** Stéphane J. Montuelle, Rachel A. Olson, Hannah Curtis, Sophia Beery, Susan H. Williams

**Affiliations:** 1 Department of Biomedical Sciences, Ohio University Heritage College of Osteopathic Medicine, Warrensville Heights, Ohio, United States of America; 2 Department of Biologic & Materials Sciences & Prosthodontics, University of Michigan School of Dentistry, Ann Arbor, Michigan, United States of America; 3 Department of Biological Sciences, Ohio University, Athens, Ohio, United States of America; 4 Department of Biomedical Sciences, Ohio University Heritage College of Osteopathic Medicine, Athens, Ohio, United States of America; Monash University, AUSTRALIA

## Abstract

Chewing is a rhythmic oral behavior that requires constant modifications of jaw movements in response to changes in food properties. The food-specific kinematic response is dependent on the potential for kinematic flexibility allowed by morphology and modulation of motor control. This study investigates the effects of food toughness and stiffness on the amplitude and variability of jaw movements during chewing in a typical omnivorous mammalian model (pigs). Jaw movements were reconstructed using X-ray Reconstruction Of Moving Morphology (XROMM) and kinematic data associated with the amplitude of jaw pitch (opening-closing) and jaw yaw (mediolateral rotation) were extracted for each cycle. Between-food differences were tested for the amplitude of jaw movements during each phase of the gape cycle, as well as in their respective within-food variability, or stereotypy, as indicated by coefficients of variation. With increasing toughness, jaw pitch amplitude is decreased during fast close, larger and more stereotyped during slow close, smaller but more variable during slow open, and more variable during fast open. In addition, when chewing on tougher foods, the amplitude of jaw yaw during slow close only increases in a subset of individuals, but all become less variable (i.e., more stereotyped). In contrast, increasing food stiffness has no effect on the amplitude or the variability of jaw pitch, whereas jaw yaw increases significantly in the majority of individuals studied. Our data demonstrate that food stiffness and toughness both play a role in modulating gape cycle dynamics by altering the trajectory of jaw movements, especially during the slow-close phase and tooth-food-tooth contact, albeit differently. This highlights how a generalist oral morphology such as that of pigs (e.g., bunodont teeth lacking precise occlusion, permissive temporomandibular joint allowing extensive condylar displacements in 3 dimensions) enables organisms to not only adjust chewing movements in their amplitude, but also in their variability.

## 1. Introduction

In mammals, smooth and controlled jaw movements during mastication are the result of a centrally (i.e., brainstem) generated motor program coordinating jaw-opening and closing muscles. This motor pattern is then modified in response to changes in the physical and material properties of the food being processed [e.g, 1–4]. The capacity for some omnivorous mammalian species to efficiently chew different foods may be dependent on the flexibility of the jaw muscles activation pattern (sensu [5]; i.e., variability in response to change in treatment or stimulus, here food properties) to alter jaw movements during chewing in such a way that changes the interaction between the food and the oral structures involved, including the teeth for initiating and propagating food fracture and the tongue for positioning, manipulating and transporting the food bolus in the oral cavity. This flexibility can ultimately result in changes in the duration amplitude and/or velocity of jaw movements [6–8]. When the duration of movement increases, the amplitude of movement may increase, stay the same, or decrease depending on whether there are concomitant changes in velocity. Moreover, any of these changes may manifest at the level of the whole gape cycle and/or within particular gape cycle phases.

There is ample evidence across mammals documenting that different foods alter the jaw movements that occur during chewing [e.g., 9–11], with most comparing between “hard” and “soft” foods, often at extreme ends of a texture range. However, the extent to which specific kinematic strategies occur in relation to measured mechanical properties has only been systematically investigated for a few mammalian species [6, 8, 12–14]. These studies are important because they not only provide a better understanding of the dynamic tooth-food interaction in the context of fracture mechanics, which is essential for linking tooth morphology with function, but also of the factors that influence neuromotor control, in this case, the food properties that significantly alter the motor control of chewing movements. For example, Reed and Ross [8] demonstrated that in the primate *Cebus*, foods of high toughness are associated with large vertical jaw displacements during chewing, whereas foods of low toughness are associated with large horizontal displacements. Moreover, *Cebus* maintain gape cycle duration when feeding on tough foods, but alter the duration and amplitude of jaw movements within each cycle. In addition, when compared to *Macaca*, *Cebus* gape cycles exhibit overall higher variability in the temporal and spatial dynamics of their jaw movements, particularly when chewing on foods of low toughness [6]. These differences between species may be associated with differences in their ability to modulate jaw movements, their morphology (e.g., compared to *Macaca*, *Cebus* have relatively flat occlusal surfaces), and/or differences in intraoral bolus manipulation and partitioning behaviors. Thus interspecific differences in masticatory flexibility may have underlying neuromotor, morphological, and/or behavioral correlates.

In pigs, also omnivorous, Herring and colleagues demonstrated that jaw movements during chewing adjust to the changes in food properties as well, even in young individuals with a mixed dentition containing deciduous teeth [e.g., 15–19]. For example, hard foods tend to yield longer gape cycles due to longer power strokes and more lateral deviation of the jaw with decreased vertical jaw opening, whereas soft foods elicit greater jaw pitch during opening [16,18]. More recently, Menegaz et al. [19] showed that jaw movements during the initial cracking of a hard nut emphasizes the vertical component of jaw rotation (i.e., jaw pitch), but that during the reduction stage (i.e., once fracture had occurred), jaw movements are more similar to those used to process less-resistant foods. While the sample sizes of jaw movements in these studies limits a full analysis of variability between- and within-food, they indicate that there are likely consistent changes associated with the mastication of foods of different properties.

Previously, we showed that the temporal dynamics of pig jaw movements are significantly modified in response to changes in both food toughness and stiffness, albeit differently [7]. Toughness is a measure of the energy release rate, or the energy needed to propagate a crack, whereas stiffness (i.e., the elastic modulus) represents resistance to elastic deformation [20–22]. An increase in food toughness increases gape cycle duration whereas an increase in food stiffness decreases gape cycle duration. These contrasting strategies for dealing with changes in food toughness *versus* stiffness highlight the inherent flexibility of the pig masticatory system.

Although it is reasonable to expect substantial differences in jaw movements between foods of different properties, several studies have also demonstrated that kinematic differences within the same foods account for a significant component of variability in masticatory dynamics [e.g., 8, 23]. In other words, mammalian chewing movements show high levels of variability within a particular treatment or stimulus and thus may not be stereotyped (sensu 5). This means that jaw movements can vary from one cycle to the next within a sequence while the food bolus is being processed. This has been demonstrated directly for pigs and primates, which show differences in the duration of gape cycle parameters within a food [7, 8]. Moreover, the level of stereotypy exhibited during chewing depends on the properties of the food item. Species with high levels of stereotypy exhibit repeatable jaw movements whereas those with low levels exhibit more variable movements in response to the same food. Constant adjustments of chewing movements (i.e., less stereotyped and more variable movements) may be disadvantageous if they increase the energy required to chew food and/or increase tooth wear or the potential for damage [24–26]. Previously, we found that the duration of slow closing, during which most of the tooth-food-tooth contact occur, becomes more stereotyped (i.e., less variable) with an increase in food toughness and stiffness. Thus the inherent flexibility of jaw movement during chewing in pigs may ultimately be constrained if certain food properties require more adjustable jaw movements from one chewing cycle to the next.

In the present study, we investigate how changes in food toughness and stiffness alter the amplitude of jaw pitch and yaw movements, and their variability, during chewing in pigs to understand whether, and if so, how previously observed flexibility and stereotypy in the timing of jaw movements translates into changes in their spatial dynamics. We predict that stiffer foods will elicit smaller and less variable jaw movements reflecting more precise control of the jaw during chewing. This may also reflect an increased need to coordinate jaw and tongue movements during chewing because stiff foods may suddenly fracture. Because pigs are morphologically well-suited for crushing and grinding but lack the prominent serially-arranged shearing crests of herbivores required for fracturing tough foods, we predict that mastication of tough foods will require larger amplitude and more variable jaw movements, especially during tooth-food-tooth or tooth-tooth contact during slow closing and the occlusal phase in particular.

## 2. Material & methods

### 2.1. Study design and data collection

The present study utilizes much of the same data collected for our previously published study on the effect of toughness and stiffness on temporal dynamics of jaw movements [7] in which animals were fed 3 foods (carrots, apples and almonds) that mainly differ in one food property while being comparable in the other property. Toughness and stiffness data were based on previously published values [27]. Of the three foods, apples have the lowest toughness and stiffness whereas almonds have the highest toughness and stiffness. Carrots have a comparable toughness to almonds but are not as stiff (see Table 1). One caveat in the study design is that these foods are a mosaic of different material properties and thus our comparisons may not correspond to a direct test of the effects of changes in one single material property. Nevertheless, bivariate comparisons between the three foods allows for testing the effects of food toughness while minimizing the effects of changes in food stiffness (i.e., apples *versus* carrots), and for testing the effects of food stiffness while minimizing the effects of food toughness (i.e., almonds *versus* carrots). Foods were offered in pieces of similar size (approximately 2 cm x 2 cm x 1 cm) to control for the effects of food size, but other food properties (e.g., color, smell) were not controlled and may therefore have some effects.

**Table 1:** Sample sizes, measurement errors and precision thresholds for each animal.

	Number of chews						Measurement error (of rigid body motion)		Precision threshold (for movement detection)		
	Apple		Carrot		Almond		Skull beads	Jaw beads	Ry	Rz	Tx
Animal ID	Left	Right	Left	Right	Left	Right					
5	35	24	50	42	50	50	0.14 mm	0.17 mm	0.13°	0.10°	0.07 mm
6	34	42	38	45	5	6	0.73 mm	0.70 mm	0.08°	0.12°	0.06 mm
9	76	75	67	71	107	128	0.47 mm	0.47 mm	0.47°	0.21°	0.01 mm
10	79	72	4	6	156	146	0.45 mm	0.38 mm	0.10°	0.12°	0.09 mm

Measurement error is quantified by the average standard deviation of the 3D distance between markers implanted in the same rigid body (i.e., skull or jaw). Precision thresholds for movement detection for Ry, Rz and Tx (see Material and methods for more details).

Foods were presented to the animal in a bowl allowing them to feed ad libitum. This helped keep the animals within the field of view of the fluoroscopes and the animals could control how much they ingested. Animals most often ingested one piece at a time, but they could ingest then chew for sequential cycles and ingest another piece adding it to the bolus. As a result, within a single feeding bout, pigs tend to ingest, chew and swallow continuously, and the feeding sequences recorded often included ingestion and swallowing cycles randomly inserted among a series of chewing cycles. This prevented us from sequence level analysis and chew-per-bolus analysis (e.g., 6, 8). Ingestion and swallowing cycles were removed from the dataset so that the analysis only includes the gape cycles corresponding to chewing.

The study design and methods are identical to our previous analysis of the timing of jaw movements during pig chewing [7]. Jaw movements during pig chewing were quantified using marker-based X-ray Reconstruction Of Moving Morphology (XROMM) [28] in four 3-to-4-month-old female Hampshire cross-breed pigs (*Sus scrofa* Linnaeus 1758) weighing between 14–20 kg. The skull and jaw of each animal were implanted with 5 to 6 radiopaque tantalum beads (1 mm diameter, Bal-Tec, Los Angeles, CA, USA) while under isoflurane anesthesia. Feeding videos were recorded daily using two synchronized high-speed cameras (Oqus 310, Qualisys, Göteborg, Sweden) mounted on the output ports of two fluoroscopes (OEC-9000). At the beginning and at the end of each recording session, the field of view was calibrated using (i) a perforated steel sheet with standardized hole spacing and size (item #9255T641, McMaster-Carr, Robinson, NJ,USA) to correct for distortion inherent to x-ray imaging, and (ii) a custom-built cube of 4 plastic sheets containing 64 radiopaque tantalum beads (placed in a 4 × 4 fashion 2.5 cm apart from one another) to calculate the position of each fluoroscope relative to each other.

Over the course of data collection, animals were CT-scanned to produce the 3D bone models necessary for the XROMM methodology [28]. In total, the animals were CT-scanned three times: once after implantation surgery, once midway through data collection and once after euthanasia once a sufficient dataset was recorded. *In vivo* scanning was performed under isoflurane anesthesia at The Ohio State University College of Veterinary Medicine (Columbus, OH, USA) on a GE Lightspeed Ultra CT scanner. Post-mortem scanning was done locally at Holzer Clinic (Athens, OH, USA) on a Philips Brilliance 64 scanner. Animal husbandry as well as the surgical and experimental procedures were carried out in accordance with the recommendations in the Guide for the Care and Use of Laboratory Animals of the National Institutes of Health. The protocol was approved by the Ohio University Institutional Animal Care and Use Committee (Protocol 12-U-009).

### 2.2. Kinematic dataset

**Fig 1:**
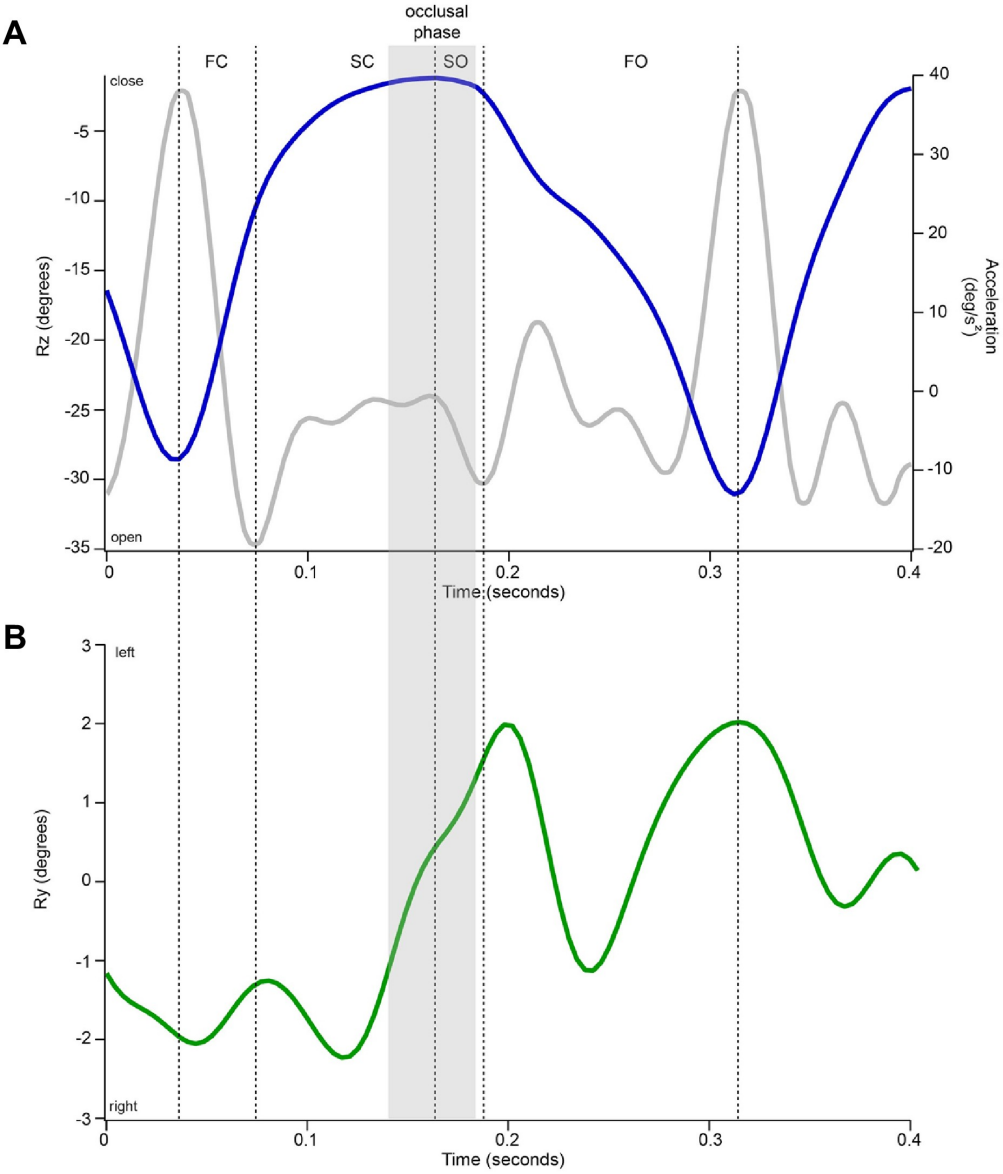
Representative traces of Rz and Ry during a single chew illustrating the kinematic variables calculated. **A**) Variation in jaw pitch over time (Rz; in blue) was used to define the start and endpoint of each gape cycle within a feeding bout by identifying the sequential time to maximum gape opening. The end of jaw and slow closing (SC) was set at the instant of minimum gape. Jaw pitch acceleration (i.e., second derivative of Rz over time; in grey) was used to identify phase transitions (i.e., FC-SC and SO-FO). The amplitude of jaw closing and opening were calculated for each phase in absolute and relative values (degrees and % of total jaw opening or closing, respectively). **B**) The absolute amplitude of jaw yaw (Ry; in green) during SC was calculated as the difference between the medial-most rotational position of the lower jaw at the end of SC and the lateral-most position observed at the beginning of SC. In this particular example, jaw yaw increases during SC, reflecting that the jaw rotates laterally towards the left which is indicative of a chew on the right-hand side of the animal. In both panels, the grey shaded area represents the occlusal phase determined visually from the Maya animations.

Using the XROMM workflow and XMALab software [29], the 3D coordinates of embedded markers were extracted and used to calculate the rigid body motions of the skull and jaw. Each chewing sequence recorded was animated frame by frame in Maya (Autodesk Inc., San Rafael, CA, USA) by assigning the rigid body motions to the corresponding 3D models of the skull and jaw (generated from computed tomography scans of each animal). A joint coordinate system (JCS) allowed quantification and comparison of the 6 degrees of freedom of jaw movements during chewing in pigs: 3 rotations (Rx, Ry, Rz) and 3 translations (Tx, Ty, Tz). For each sequence, individual gape cycles and their constituent phases were identified based on the changes in acceleration of the depression-elevation of the jaw with respect to the skull as well as jaw yaw (Fig 1). We restricted our analysis of movement to the rotational degrees of freedom that exceed our precision thresholds for quantifying biological motion during a chewing cycle (see Table 1).

In accordance with previous XROMM studies of pig chewing [28, 30], the primary degree of freedom is jaw pitch or the opening-closing movements that define each chewing cycle (i.e., Rz). Based on the Rz wave, we measured the amplitude of jaw opening and closing movements during each of the traditional phase of the gape cycle: fast close (FC), slow close (SC), slow open (SO) and fast open (FO) (Fig 1A). We also calculated the relative amplitude of FC and SC during jaw closing and the relative amplitude of SO and FO during jaw opening as a percent of total jaw closing or opening, respectively. This dataset allows us to understand trade-offs in movement between phases and the relationship with previously observed flexibility in the temporal dynamics [7].

The second largest degree of freedom in our dataset is jaw yaw (Ry), representing rotation about a dorsoventral axis. Jaw yaw is an essential component of food breakdown as it allows the food to be crushed as the lower and upper teeth slide against one another. We calculated the absolute amplitude of Ry during SC as the difference between the medial-most rotational position of the lower jaw at the end of SC and the lateral-most position recorded at the beginning of SC (Fig 1B). In addition, the animation of each chewing cycle analyzed was visually inspected to detect tooth occlusion. In chewing cycles where tooth occlusion occurred, the start and end frame of tooth occlusion were extracted and within that window, the absolute amplitude of jaw yaw during occlusion was calculated similarly to that of jaw yaw during SC. Note that in some cycles, the occlusal phase started during SC but lasted through a portion of SO (see Fig 1A). Because not every cycle had a definitive occlusal phase, the total dataset of 1408 cycles was reduced to 1206 cycles for the analysis of the occlusal phase. For both jaw yaw during SC and during occlusion, we used the absolute amplitude because left side chews are identified by a decrease in Ry whereas right side chews are identified by an increase in Ry.

Note that in accordance with previous XROMM analyses of jaw movements during pig chewing [28, 30], jaw pitch (Rz) and jaw yaw (Ry) were the only two rotational degrees of freedom detected in our dataset. Changes in the third potential rotational degree of freedom—jaw roll, (Rx, rotation of the jaw around the anteroposterior axis)–were less than the corresponding precision threshold and therefore could not be confidently interpreted as biological motion.

To quantify within-food variability (i.e., stereotypy), we calculated the coefficients of variation (CVs) for the absolute Rz amplitude corresponding to each phase and for the Ry amplitude (during SC and during occlusion) with all individuals pooled together as well as for each individual separately. Low CVs indicate stereotyped amplitudes whereas high CVs indicate variable amplitudes. A summary of the structure of the dataset, including the numbers of gape cycles analyzed per animal and per food, is provided in Table 1.

### 2.3. Statistical analysis

Between-food variability (i.e., flexibility sensu 5) was examined for all kinematic variables using a linear mixed model fit analysis of variance coupled with t-tests and univariate F-ratio’s. Similarly, to determine whether within-food variability differs between foods, the CVs associated with each food and individual were analyzed using the same method. In both cases, a first analysis tested the effects of changes in food toughness while minimizing the effects of changes in stiffness (i.e., apple *versus* carrot), whereas the second analysis tested the effects of changes in food stiffness while minimizing the effects of changes in toughness (i.e., carrot *versus* almond). In all analyses, food was entered as the fixed factor, individual and chewing side as random factors (hence the mixed model fit). Note that the associated interaction terms (e.g., Food x Side x Individual or Food x Individual interaction terms) were also entered in the initial design, but removed from the final design if not significant. The side factor and the associated interaction terms were included *a priori* to ensure that all sources of variance were considered, but were, in most cases, non-significant. In addition, because the subsamples differ in their respective variance, the analyses were conducted using Satterthwaite's approximation of the pooled variance. All statistical analyses were performed using the lmer function from the lme4 package in R Studio (Boston, MA, USA).

## 3. Results

### 3.1. Effects of food properties on the amplitude of jaw movements

**Fig 2:**
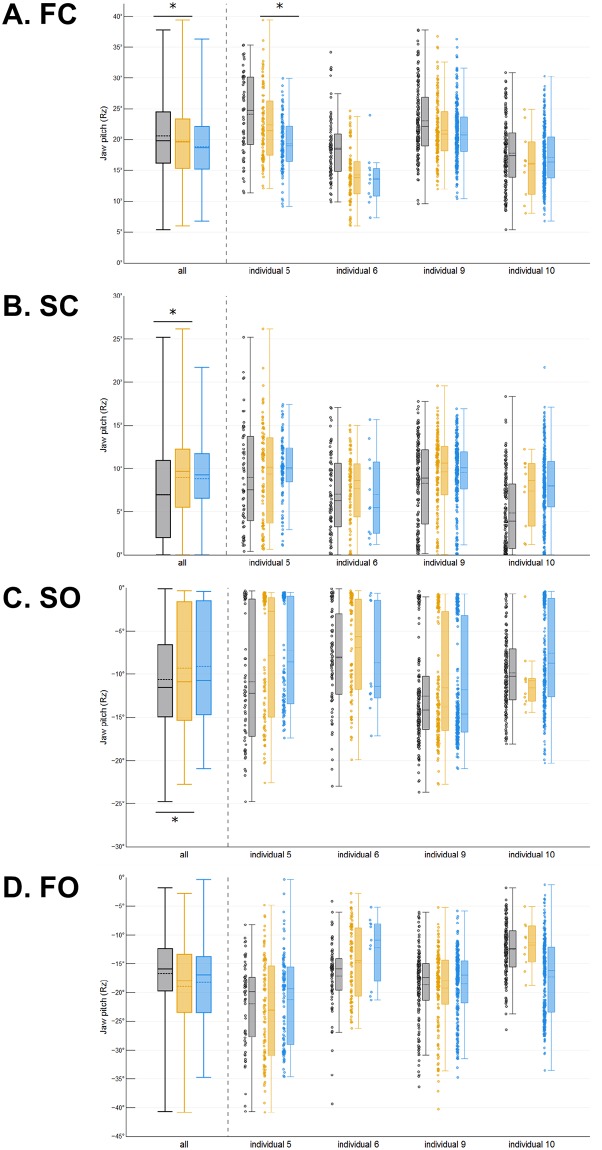
Effect of food toughness and stiffness on the amplitude of jaw pitch (Rz). Plots illustrate the absolute amplitude of jaw closing during each phase of the gape cycle for each food: **A**) FC, **B**) SC, **C**) SO and **D**) FO. Colors represent foods: apples in black, carrots in orange and almonds in blue. Each box is defined by the 25^th^ and 75^th^ quartiles as its lower and upper limit, respectively. In each box, the solid line is the median, the dashed line is the mean, and whiskers indicate the 5^th^ and 95^th^ percentiles. Individual data points are shown next to each box. Significant differences between foods are indicated by an *.

Table 2 provides the means and standard errors for each variable for each food, while the results of the analyses of variance evaluating the effect of toughness and stiffness on the amplitude of jaw movements are presented in Tables 3 and 4, respectively. These results are illustrated in Figs 2 through 4.

**Fig 3:**
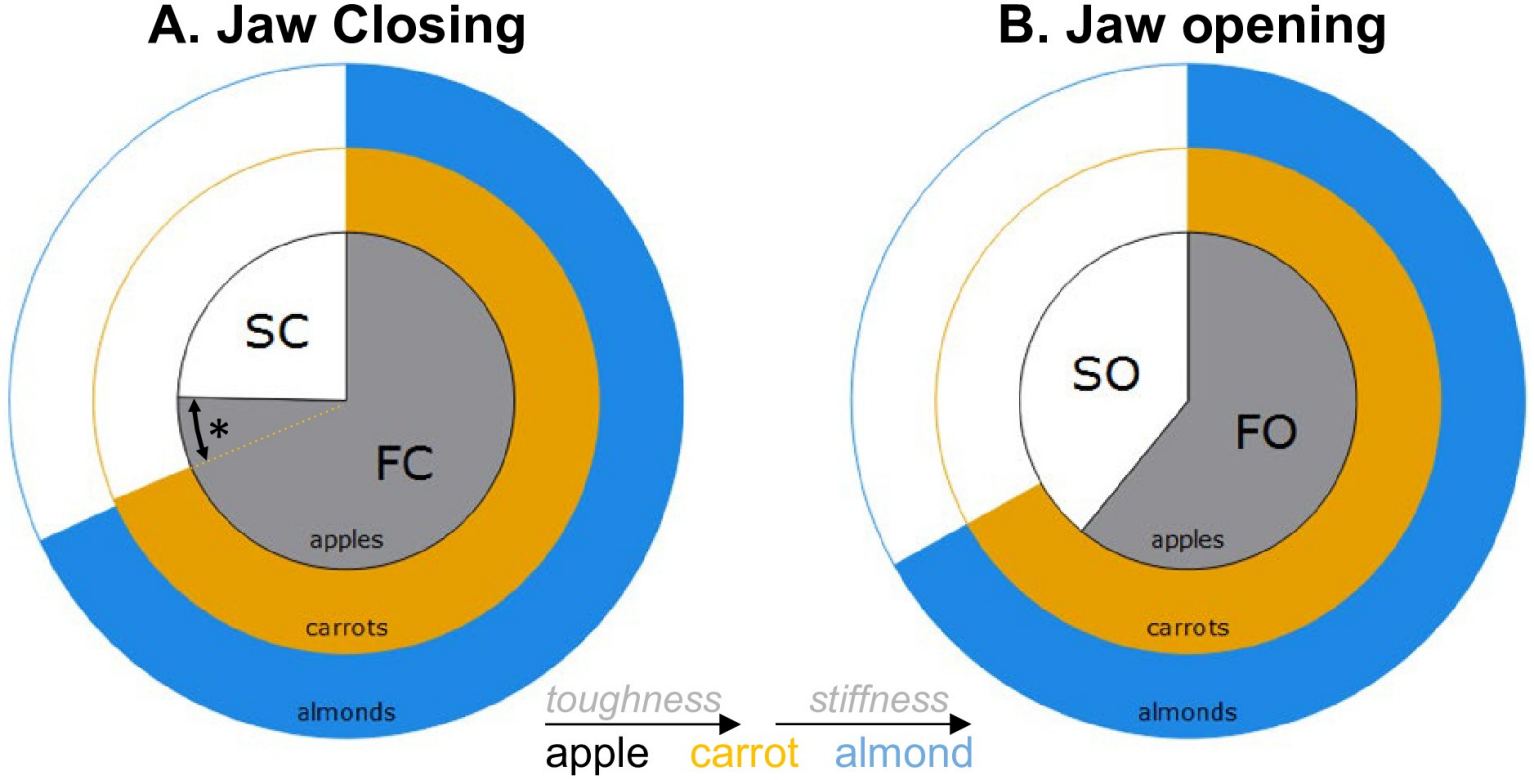
Effect of food toughness and stiffness on the relative amplitude of jaw pitch (Rz). Pie charts illustrate the relative amplitude of (**A**) jaw closing during FC and SC and (**B**) jaw opening during SO and FO. Relative amplitudes are expressed as a % of total amplitude of either jaw opening or closing. Colors represent foods: apples in black, carrots in orange, and almonds in blue. Significant differences between foods are indicated by an *.

**Fig 4:**
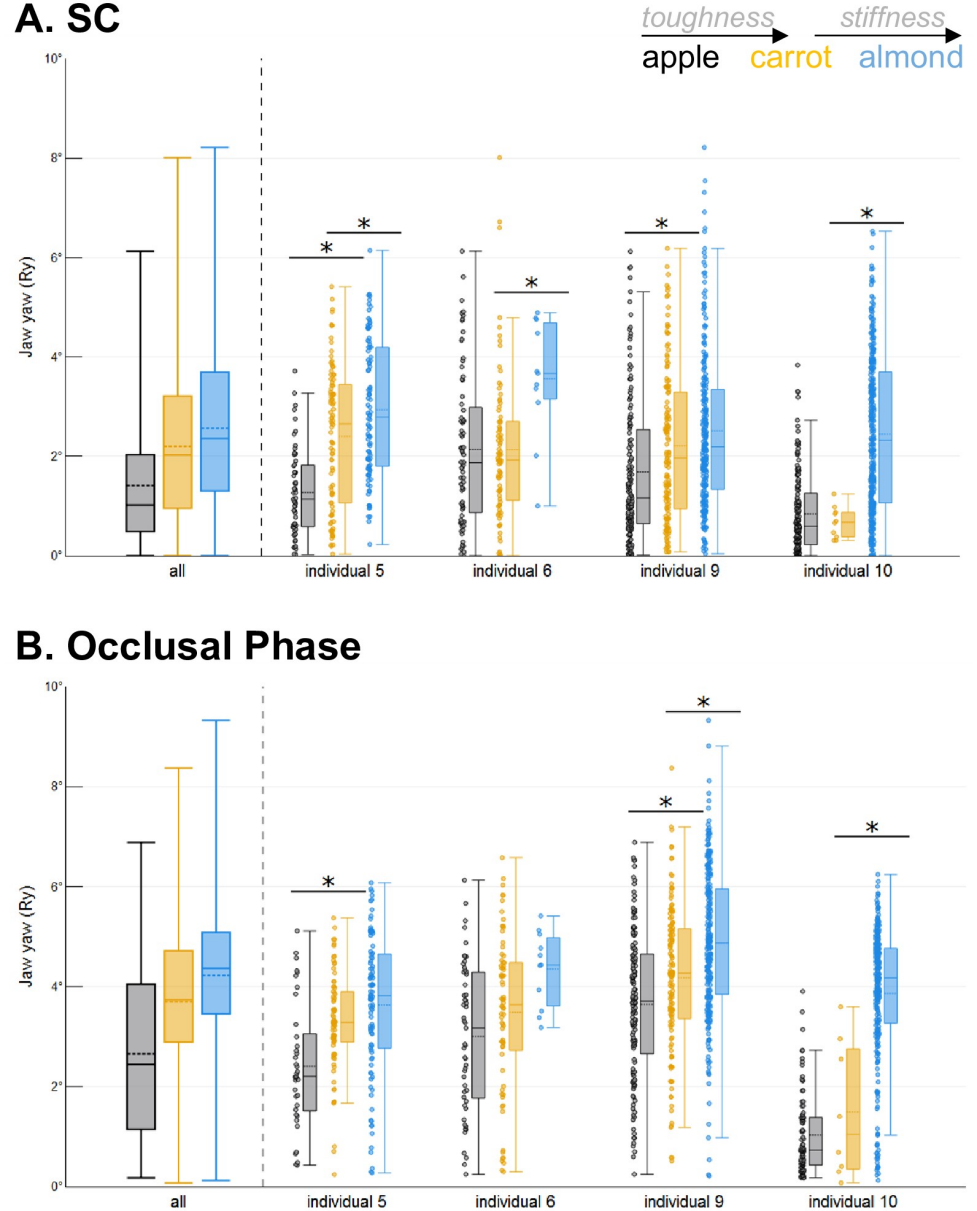
Effect of food toughness and stiffness on the amplitude of jaw yaw (Ry). Plots illustrate the absolute amplitude of jaw yaw for each food: **A**) during SC and **B**) during the occlusal phase. Colors represent foods: apples in black, carrots in orange and almonds in blue. Each box is defined by the 25^th^ and 75^th^ quartiles as its lower and upper limit, respectively. In each box, the solid line is the median, the dashed line is the mean, and whiskers indicate the 5^th^ and 95^th^ percentiles. Individual data points are shown next to each box. Significant differences between foods are indicated by an *.

**Table 2:** Summary data for jaw movements in pigs chewing on food varying in toughness and stiffness.

Food	Apple			Carrot			Almond		
Toughness	56.97 ± 17.76 J.m^-2^			343.93 ± 48.49 J.m^-2^			308.62 ± 34.85 J.m^-2^		
Stiffness	3.41 ± 0.10 MPa			6.86 ± 0.46 MPa			19.42 ± 7.69 MPa		
	All chews	Right chews	Left chews	All chews	Right chews	Left chews	All chews	Right chews	Left chews
	N = 402	N = 185	N = 217	N = 375	N = 183	N = 192	N = 631	N = 312	N = 319
**Elevation (Rz)**									
FC (deg)	12.9 ± 0.2	12.6 ± 0.3	13.4 ± 0.3	12.2 ± 0.2	12.1 ± 0.3	12.2 ± 0.3	12.0 ± 0.1	12.0 ± 0.2	12.0 ± 0.2
SC (deg)	3.5 ± 0.1	3.6 ± 0.2	3.6 ± 0.2	4.2 ± 0.1	4.2 ± 0.2	4.2 ± 0.2	4.0 ± 0.1	3.9 ± 0.1	4.0 ± 0.1
FC (%)	78.5 ± 0.7	78.0 ± 1.1	78.8 ± 1.0	73.7 ± 0.7	73.7 ± 1.1	73.6 ± 1.0	75.3 ± 0.5	75.9 ± 0.7	74.8 ± 0.7
SC (%)	21.5 ± 0.7	22.0 ± 1.1	21.2 ± 1.0	26.3 ± 0.7	26.3 ± 1.1	26.4 ± 1.0	24.7 ± 0.5	24.1 ± 0.7	25.2 ± 0.7
**Depression (Rz)**									
SO (deg)	6.1 ± 0.1	6.1 ± 0.2	6.4 ± 0.2	7.0 ± 0.1	6.9 ± 0.2	7.2 ± 0.2	6.6 ± 0.1	6.7 ± 0.1	6.5 ± 0.1
FO (deg)	10.1 ± 0.2	10.1 ± 0.3	10.4 ± 0.3	9.3 ± 0.2	9.5 ± 0.3	9.0 ± 0.3	9.4 ± 0.1	9.4 ± 0.2	9.3 ± 0.2
SO (%)	38.1 ± 0.6	38.1 ± 1.0	38.3 ± 0.8	44.1 ± 0.7	42.7 ± 1.0	45.5 ± 1.1	42.3 ± 0.5	42.3 ± 0.7	42.2 ± 0.8
FO (%)	61.9 ± 0.6	62.0 ± 1.0	61.7 ± 0.8	55.9 ± 0.7	57.3 ± 1.0	54.5 ± 1.1	57.7 ± 0.5	57.7 ± 0.7	57.8 ± 0.8
**Yaw (Ry) during SC (deg)**	2.2 ± 0.1	2.3 ± 0.1	2.0 ± 0.1	2.8 ± 0.1	2.9 ± 0.1	2.7 ± 0.1	3.3 ± 0.1	3.6 ± 0.1	3.1 ± 0.1
**Yaw (Ry) during occlusion (deg)**	2.7 ± 0.6	2.6 ± 0.6	2.8 ± 0.7	3.7 ± 0.4	3.6 ± 0.4	3.9 ± 0.4	4.2 ± 0.4	4.1 ± 0.4	4.3 ± 0.4

Table entries are mean ± standard error of the mean (s.e.m). Toughness and stiffness data are from [27].

**Table 3:** Summary of the effects of food toughness (i.e., apple versus carrot) on the amplitude of jaw movements.

	Interaction terms			Differences among individuals	Differences between Foods	
	F x I x S	F x S	F x I		All individuals	At the individual level
**Elevation (Rz)**						
FC (deg)	NS	NS	NS	***	*F*_1,759_ = 26.80***	-
SC (deg)	NS	NS	NS	***	*F*_1,753_ = 8.36**	-
FC (%) and SC (%)	NS	NS	NS	*	*F*_1,543_ = 21.97***	-
**Depression (Rz)**						
SO (deg)	NS	NS	NS	***	*F*_1,748_ = 8.36**	-
FO (deg)	NS	NS	NS	***	NS	-
SO (%) and FO (%)	NS	NS	NS	***	NS	-
**Yaw (Ry)**						
during SC (deg)	NS	NS	*	-	-	5: *F*_1,152_ = 28.40***
						9: *F*_1,285_ = 9.38***
						6 and 10: NS
during occlusion (deg)	NS	NS	*	-	-	5: *F*_1,110_ = 15.76***
						9: *F*_1,288_ = 8.87**
						6 and 10: NS

F, food; I, individual; S, side. Non-significant interaction terms were removed from the final design, whereas significant interaction terms prompted further analyses to be conducted at the individual level. Table entries are the univariate F-ratio;

* indicates 0.050 > P > = 0.010,

** indicates 0.010 > P ≥ 0.001, and

*** indicates P < 0.001.

**Table 4:** Summary of the effects of food stiffness (i.e., carrot versus almond) on the amplitude of jaw movements.

	Interaction terms			Differences among individuals	Differences between Foods	
	F x I x S	F x S	F x I		All individuals	At the individual level
**Elevation (Rz)**						
FC (deg)	NS	NS	*	-	-	5: *F*_1,191_ = 17.65***
						6, 9 and 10: NS
SC (deg)	NS	NS	NS	***	NS	-
FC (%) and SC (%)	NS	NS	NS	NS	NS	-
**Depression (Rz)**						
SO (deg)	NS	NS	NS	***	NS	-
FO (deg)	NS	NS	NS	***	NS	-
SO (%) and FO (%)	NS	NS	NS	***	NS	-
**Yaw (Ry)**						
during SC (deg)	NS	NS	*	-	-	5: *F*_1,191_ = 6.79**
						6: *F*_1,94_ = 9.53**
						10: *F*_1,312_ = 12.98***
						9: NS
during occlusion (deg)	NS	*	*	-	-	9^R^: *F*_1,198_ = 32.63***
						10: *F*_1,273_ = 24.50***
						5, 6 and 9^L^: NS

F, food; I, individual; S, side. Non-significant interaction terms were removed from the final design, whereas significant interaction terms prompted further analyses to be conducted at the individual level. Table entries are the univariate F-ratio;

* indicates 0.050 > P > = 0.010,

** indicates 0.010 > P > = 0.001, and

*** indicates P < 0.001.

*Jaw pitch (Rz)*. The absolute amplitude of jaw pitch during FC, SC and SO and the relative amplitude of FC and SC differed between carrot and apple (Table 3). When chewing carrots, as compared to apples, jaw movements in the pigs are characterized by greater Rz amplitude during SC (Figs 2B and 3A) but smaller amplitude during FC and SO (Fig 2A and 2C). In contrast, increased food stiffness had a very limited effect on the amplitude of jaw pitch (Table 4 and Fig 3). Only one individual (individual 5) had a significantly smaller closing amplitude during FC when chewing on almonds, compared to carrots (less stiff; Fig 2A). Note that these significant effects were accompanied by significant differences among individuals (Tables 3 and 4).

*Jaw yaw (Ry)*. Both the amplitude of jaw yaw during SC and the occlusal phase were characterized by significant Food x Individual interactions (Tables 3 and 4), indicating that changes in food properties only apply to a subset of our dataset. First, jaw yaw during SC and the occlusal phase was significantly greater when chewing on carrots as compared to apples (less tough) in 2 of the 4 individuals observed (individuals 5 and 9; Fig 4A and 4B). Second, jaw yaw during SC was significantly greater when chewing on almonds as compared to carrots (less stiff) in 3 of the 4 individuals (individuals 5, 6 and 10; Fig 4A). Finally, the effects of food stiffness were only significant for the amplitude of jaw yaw during the occlusal phase in 2 individuals (individual 10 and the right chews of individual 9; Fig 4B).

### 3.2 Effects of food properties on the variability of jaw movements

**Fig 5:**
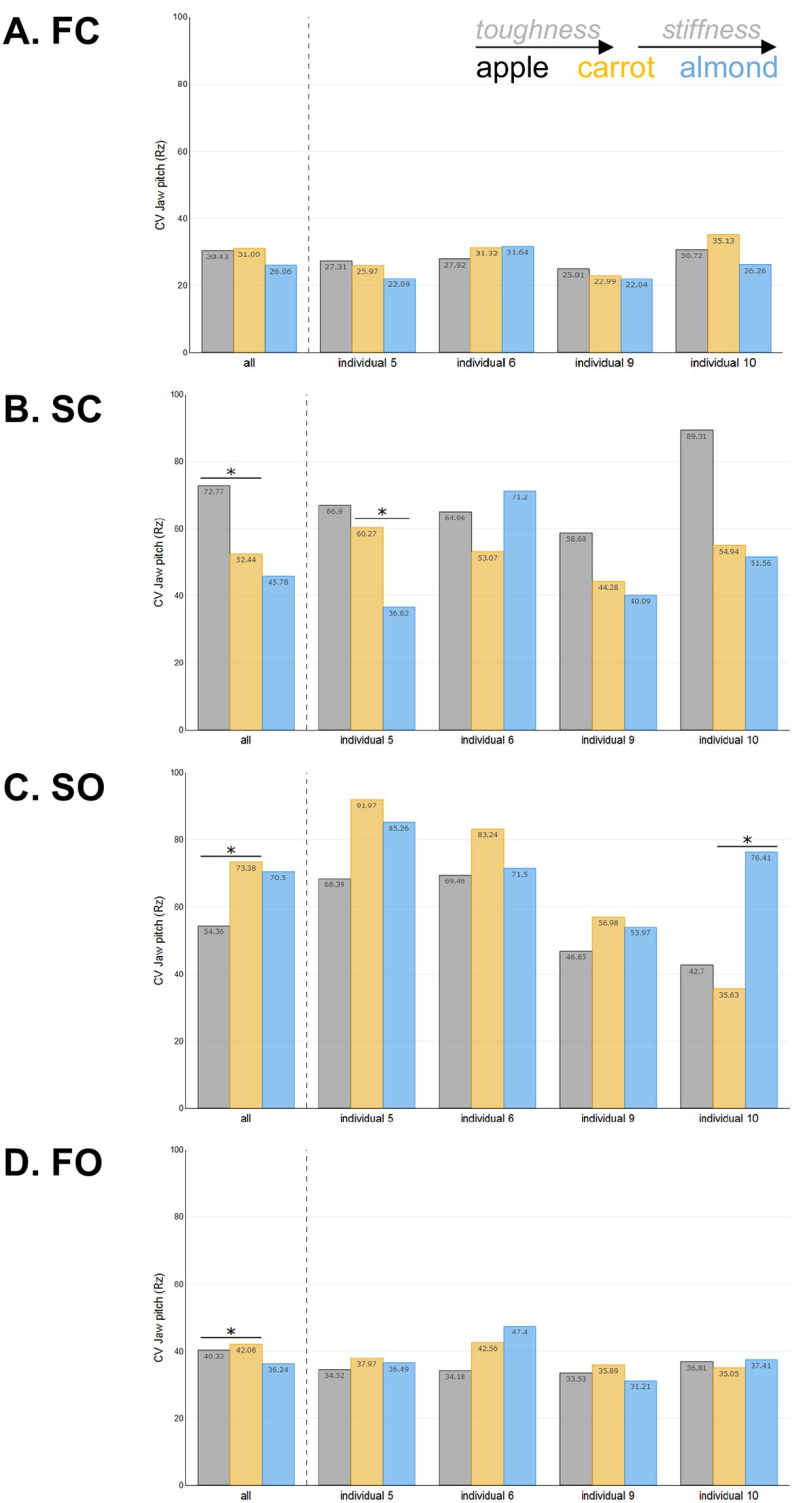
Effect of food toughness and stiffness on the within-food variability of jaw pitch amplitude. CV indicates the level of stereotypy in the amplitude of Rz for each food during each phase: **A**) FC, **B**) SC, **C**) SO and **D**) FO. Colors represent foods: apples in black, carrots in orange and almonds in blue. Significant differences between foods are indicated by an *.

**Fig 6:**
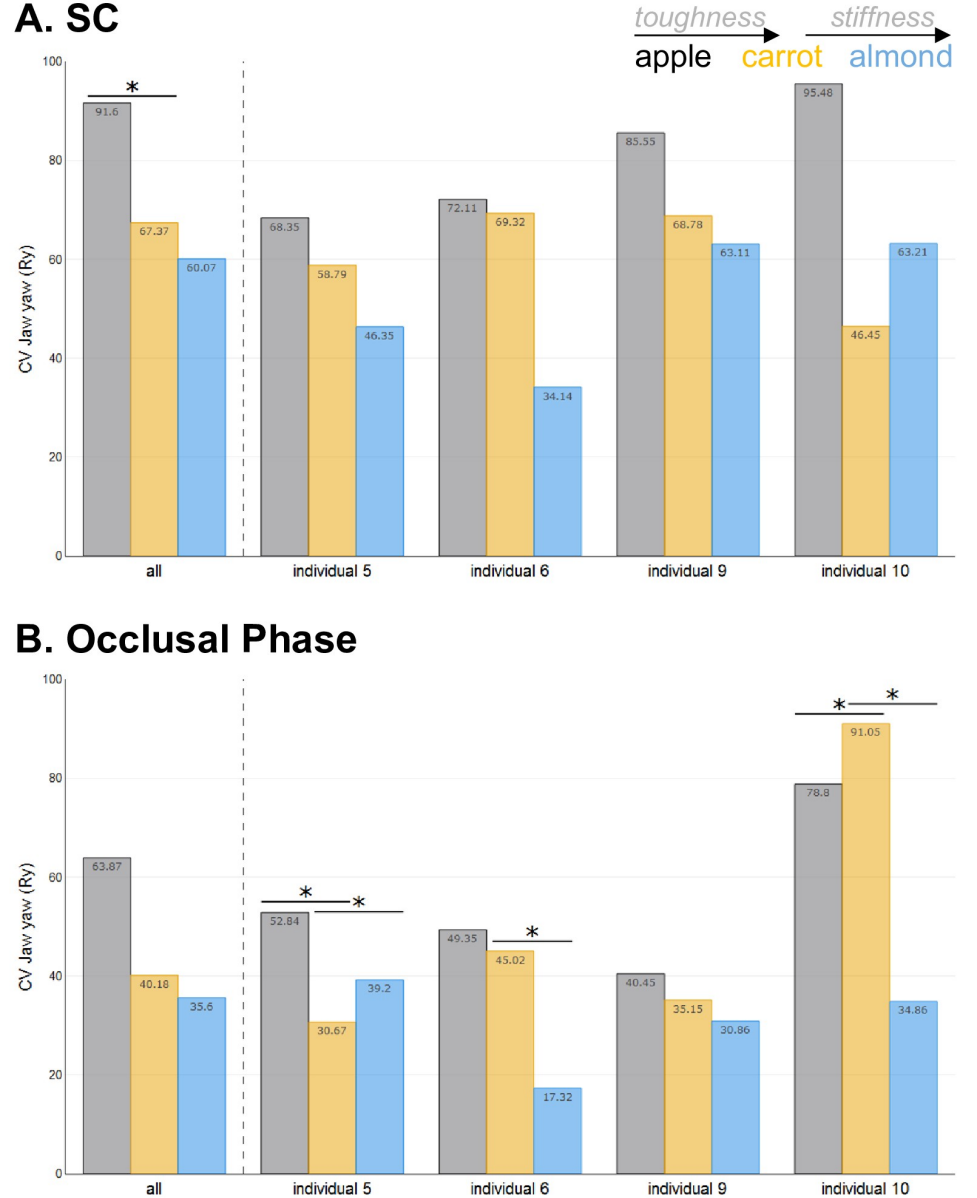
Effect of toughness and stiffness on the within-food variability of jaw yaw amplitude. CV indicates the level of stereotypy in the amplitude of Ry for each food: **A)** during SC and **B)** during tooth occlusion (when present). Colors represent foods: apples in black, carrots in orange and almonds in blue. Significant differences between foods are indicated by an *.

Tables 5 and 6 present the results of the analyses of variance evaluating the effect of toughness and stiffness on the CVs of jaw movements, respectively. These results are illustrated in Figs 5 and 6.

**Table 5:** Summary of the effects of food toughness (i.e., apple versus carrot) on the variability of jaw movements.

	Interaction terms			Differences among individuals	Differences between Foods	
	F x I x S	F x S	F x I		All individuals	At the individual level
**Elevation (Rz)**						
FC (deg)	NS	NS	NS	*	NS	-
SC (deg)	NS	NS	NS	***	*F*_1,751_ = 24.67***	-
**Depression (Rz)**						
SO (deg)	NS	NS	NS	***	*F*_1,760_ = 45.09***	-
FO (deg)	NS	NS	NS	NS	*F*_1,397_ = 8.16*	-
**Yaw (Ry)**						
during SC (deg)	NS	NS	NS	NS	*F*_1,430_ = 10.73**	-
during occlusion (deg)	*	NS	NS	NS	-	5: *F*_1,111_ = 13.83***
						10^R^: *F*_1,47_ = 14.44***
						6, 9 and 10^L^: NS

F, food; I, individual; S, side. Non-significant interaction terms were removed from the final design, whereas significant interaction terms prompted further analyses to be conducted at the individual level. Table entries are the univariate F-ratio;

* indicates 0.050 > P > = 0.010,

** indicates 0.010 > P > = 0.001, and

*** indicates P < 0.001.

**Table 6:** Summary of the effects of food stiffness (i.e., carrot versus almond) on the variability of jaw movements.

	Interaction terms			Differences among individuals	Differences between Foods	
	F x I x S	F x S	F x I		All individuals	At the individual level
**Elevation (Rz)**						
FC (deg)	NS	NS	NS	***	NS	-
SC (deg)	NS	NS	*	-	-	5: *F*_1,191_ = 34.43***
						6, 9 and 10: NS
**Depression (Rz)**						
SO (deg)	NS	NS	*	-	-	10: *F*_1,311_ = 20.56***
						5, 6, 9 and 10: NS
FO (deg)	NS	NS	NS	***	NS	-
**Yaw (Ry)**						
during SC (deg)	NS	NS	NS	**	NS	-
during occlusion (deg)	NS	NS	***	-	-	5: *F*_1,167_ = 6.33*
						6: *F*_1,83_ = 7.12**
						10: *F*_1,276_ = 37.73***
						9: NS

F, food; I, individual; S, side. Non-significant interaction terms were removed from the final design, whereas significant interaction terms prompted further analyses to be conducted at the individual level. Table entries are the univariate F-ratio;

* indicates 0.050 > P > = 0.010,

** indicates 0.010 > P > = 0.001, and

*** indicates P < 0.001.

*Jaw pitch (Rz)*. The CV of jaw pitch during FC did not differ between apples and carrots or between carrots and almonds (Tables 5 and 6; Fig 5A), indicating that the stereotypy of jaw pitch during that phase remained similar regardless of the food that was being processed. The CV for carrots and apples differed for SC, SO and FO. During SC, jaw pitch was significantly more stereotyped (i.e., less variable) for carrots compared to apples indicating that increased toughness decreases variability (Fig 5B). The effects of food stiffness on the stereotypy of jaw pitch during SC were less pronounced in that only 1 individual (individual 5) had significantly more stereotyped jaw pitch variation when chewing on almonds than when chewing on carrots (less stiff; Fig 5B). During SO, jaw pitch was significantly more variable for carrots than for apples (less tough; Fig 5C). As for SC, the effects of food stiffness on the variability of jaw pitch during SO were minor in that only 1 individual (individual 10) was significantly more variable in jaw pitch when chewing on almonds (Fig 5C). Finally, the variability of jaw pitch during FO was significantly greater when chewing on carrots compared to apples (less tough; Fig 5D), whereas it was unaffected by changes in food stiffness.

*Jaw yaw (Ry)*. Jaw yaw during SC was significantly more stereotyped (i.e., less variable) for carrots than apples (less tough) (Table 5 and Fig 6A). In contrast, the variability of jaw yaw during SC was not altered between carrots and almonds, indicating no effect of food stiffness (Table 6 and Fig 6A). During the occlusal phase, the effects of food properties on the variability of jaw yaw was affected by significant Food x Side and Food x Side x Individual interactions, requiring further analyses to be carried out at the individual level. For individual 5, jaw yaw during occlusion was more stereotyped for carrots than apples (less tough) whereas it was more variable for the right chews only of individual 10 (Fig 6B). When comparing almonds to carrot, jaw yaw during the occlusal phase was more stereotyped (less variable) in 2 of the 4 individuals observed (individuals 6 and 10), but more variable in one (individual 5).

## 4. Discussion

**Fig 7:**
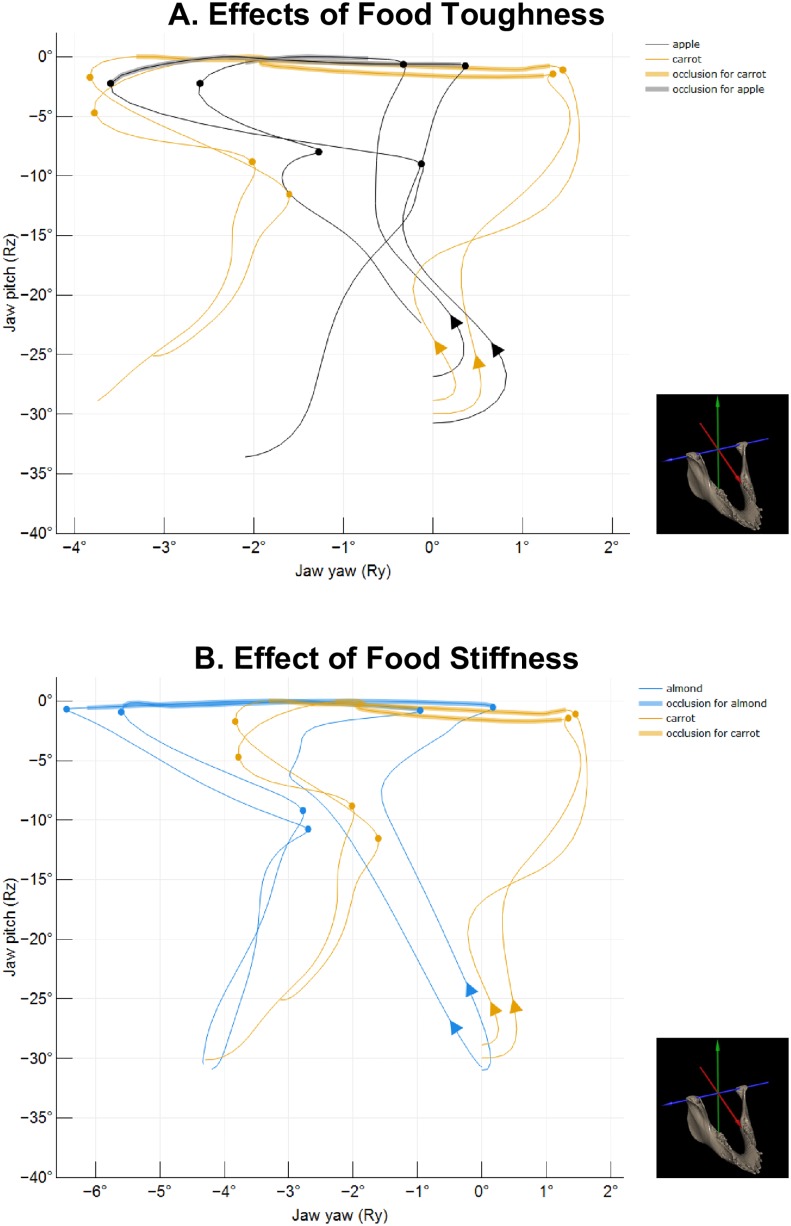
Representative chewing cycles for each food showing flexibility in pitch (Ry) versus yaw (Rz). Traces provide the trajectory of the jaw in frontal view for each food: (**A**) carrots (orange) vs apples (black) and (**B**) carrots (orange) vs almonds (blue). In A, note that the tougher food (carrot) elicits reduced Rz amplitudes during FC and an increase in Rz amplitude during SC. In B, note the overall similarity in the jaw movement profiles between carrot and almond, but that chewing stiffer food (almond) is associated with greater Ry. All traces were standardized to start at Ry = 0 and Rz was set at 0 at its minimum for that cycle. On each trace, the arrows indicate the start of each cycle, phase transitions are identified with a circle, and the occlusion phases are highlighted. Because pigs alternate chewing sides with each chew, right chews were mirrored to appear as left chews. Insets show the anatomical coordinate system used in the study.

In this study, we show that foods mainly varying in toughness and stiffness are associated with changes in the amplitude of the jaw movements during mastication in pigs. Moreover, we show that the propensity for variability of chewing movements also differs in response to toughness and stiffness. In other words, jaw movements in pigs are flexible in amplitude and in stereotypy in response to changes in food toughness and stiffness, albeit in different ways. These results reflect the modulation of motor output from the centrally-controlled motor program due to the interactions between peripheral sensory receptors in the oral cavity (e.g., periodontal mechanoreceptors) and central processing centers. The specific findings from the present study coupled with our previous work on the changes in the timing of jaw movements in response to food properties (see 7) highlight the complexity of the dynamic response of oromotor control and the potential ways that a generalized masticatory morphology may facilitate kinematic lability during chewing. Fig 7 provides an example of jaw movement profiles that encompass our general findings.

Our data show that increasing food toughness primarily results in greater emphasis on the vertical dimension of jaw movements (i.e., opening and closing) during the slow phases of the chewing cycle. This is demonstrated by the increase in the absolute and relative amplitude of jaw pitch during SC (Figs 2B and 3A), which corresponds to the portion of the gape cycle where tooth-food-tooth contact occurs. In contrast, changes in food toughness have only moderate impacts on jaw yaw as it only increased significantly in amplitude in half of the individuals studied (Fig 4). On the other hand, changes in food stiffness had little effect on the amplitude and the variability of jaw pitch, but had more effects on altering jaw yaw. Indeed, jaw yaw significantly increased when chewing on stiffer foods like almonds compared to carrots in three of the four individuals studied (Fig 4A). Based on these results, tougher foods elicit larger jaw pitch movements whereas stiffer foods are processed by using larger yaw movements.

These findings are in accordance with previous data on miniature pigs [16,18] and also mirrors that of *Cebus* [8] suggesting that at least for some taxa with bunodont cheek teeth, chewing on stiffer foods that are able to resist and sustain deformation prior to fracture utilizes more jaw yaw without a comparable change in jaw pitch. This is somewhat counterintuitive given that this pattern would have been expected in response to increased toughness because it would require the teeth to progress a crack through the material over a sustained period. This suggests that toughness may not be the sole factor influencing buccolingual movements through the occlusal phase. Nevertheless, these results are also consistent with work by Agrawal et al. [20, 31, 32] on food fragmentation and chewing. They have shown that the degree of fragmentation of 3-dimensional foods resulting from a single bite is highly correlated with the displacement-limited fragmentation index (R/E)^0.5^, where R = strain energy release rate and E = elastic modulus, a measure of stiffness. Moreover, they show a strong inverse correlation between (R/E)^0.5^ and the width of the total gape cycle and the closing angle of the jaw during SC. That is, as (R/E)^0.5^ decreases, gape cycle width and SC closing angle increase.

Using data from Williams et al. [27] for (R/E)^0.5^, the shift from carrot to almond corresponds to a decrease in (R/E)^0.5^, which is accordingly associated with the increase in buccolingual deviations during SC observed here. However, apple has the same value for (R/E)^0.5^ as almond so we would also expect it to elicit larger movements during SC. That our results only show increases in jaw closing may be due to the fact that apple may be more compliant. Compliant materials have a low stress-limited fragmentation index as well as a low displacement-limited fragmentation index (given by (E*R)^0.5^ [20, 27]. Compliant materials tolerate increased elastic deformation, absorb greater strains, and require less stress to produce larger strains. This compliance may be due to the fact that apple cells collapse or shear under compression depending on the direction of the load relative to their radially-elongate cells [33]. Thus simply approximating the teeth during closing with little to no jaw yaw, i.e., crushing, is likely sufficient to break down apple. Whether this is normal biological variation or a departure from the relationship shown by Agrawal et al. [31] is unknown. Moreover, intrinsic structural differences between carrot and almond along with potential differences in the placement of the food on the toothrow may also drive flexibility of chewing behavior in pigs, and as such may also be additional unassessed factors in our results.

Interestingly, flexibility of jaw movements during chewing in pigs is not limited to changes in their amplitude, but also in their respective within-food variability (i.e., stereotypy). In particular, during SC, jaw pitch increased and was more stereotyped when chewing on tougher foods like carrots (compared to apples; Fig 5B), whereas during SO, jaw pitch increased but was more variable (Fig 5C). Jaw pitch movements are thus more repeatable (i.e., more stereotyped and less variable) when the teeth engage with tougher foods during SC, but are less consistent when they disengage from it during SO. This may be related to jaw-tongue coordination. Indeed, as the bolus is broken down into smaller particles by the teeth, the tongue also plays a key role in gathering, manipulating and positioning the food particles. As the jaw slowly opens and the teeth disengage, the tongue protracts and deforms to collect the food particles. As such, tongue movements may have to be more responsive to the variability and unpredictability of food position during SO, therefore more variable from one cycle to the next. Consequently, jaw pitch movements may have to be more variable when chewing on tougher foods because they accommodate for more variable tongue movements.

Jaw yaw movements during SC were also less variable (i.e., more stereotyped) when chewing on tougher foods like carrots (compared to apples; Fig 6A). Reducing variability (i.e., increasing stereotypy) of jaw yaw movements increases their repeatability which highlights that fracture of tough foods may be achieved by a repeated series of precise and similar bites over the course of multiple gape cycles, rather than modulating or adjusting jaw yaw from one cycle to the next. This, coupled with the fact that jaw yaw amplitude itself is not significantly altered by food toughness, emphasizes the fact that flexibility of jaw movements in response to changes in food toughness primarily focuses on the modulation of jaw pitch movements. Increased stereotypy in jaw movements during SC is also consistent with our previous study on the timings of jaw movements which demonstrated that increasing food toughness reduced within-food variability in SC duration [7]. As the duration and/or magnitude of the force that is generated during the occlusal phase increases to process tougher foods, a less variable response occurs in the amplitude of movements. Although previous work suggests that this may be due to the need to decrease tooth wear [24–26], this is likely not an issue for our animals, who had deciduous teeth at the time of the study, nor for pigs in general, which have bunodont cheek teeth and do not rely on precise occlusion. Rather, it may be that varying the response would produce no net gain in fracture precisely because the occlusal relations between upper and lower teeth are not well-suited for driving a crack through the food using more jaw yaw. Thus breakdown may simply be a function of crushing between vertically opposed occlusal surfaces. Additionally, perhaps the range of toughness of the food used was simply not sufficient to elicit distinct kinematic responses. The fact that the enamel of pig molars are fortified against variably-oriented stresses [34] suggests that extending the range of toughness and stiffness may result in more variability both within and between foods to deal with unpredictable occlusal loads.

Finally, the occlusal morphology of pig molars changes significantly with wear allowing new or additional cusps to participate in food breakdown over the animal’s lifespan [35]. Because we only sampled young individuals without significant wear, our results apply only to a narrow window of the functional life of the deciduous dentition. Sampling jaw movements across the lifespan of the same individual would allow testing how experience and maturation contribute to their flexibility, and thus would be an insightful addition to this research. Nevertheless, given the age of our individuals, we have likely captured many of the other major changes in the feeding apparatus (e.g., masticatory muscle anatomy and activity patterns), that are associated with the ontogeny of mastication in pigs [e.g., 15–17, 36].

## 5. Conclusions

Our results indicate that both food stiffness and toughness play a role in modulating the dynamics of the gape cycle during rhythmic chewing in pigs by altering the kinematic profile of jaw movements. Increased toughness emphasizes greater and more stereotyped jaw pitch during tooth-food-tooth contact, but has limited effect on the amplitude of jaw yaw. In contrast, increased stiffness elicits greater jaw yaw movements while opening and closing movements are less affected. Such modulation of the kinematic strategy, particularly during the tooth-food-tooth contact, illustrates the mechanical requirements of breaking down a food item that is more resistant to crack propagation. Our results also highlight that flexibility of jaw movements in omnivorous mammals like pigs is not limited to altering the amplitude of jaw movements (e.g., how wide the jaw opens or deviate from the midline during the power stroke), but rather also encompasses significant changes in their variability. In particular, as food toughness increases, jaw pitch and jaw yaw during SC are more stereotyped, indicating that increasing the repeatability of jaw movements plays a key role in breaking down tough foods in animals that lack precise occlusion such as in the young pigs studied here.

## Abbreviations

CV: Coefficient of Variation
FC: Fast closing phase
FO: Fast opening phase
Ry: yaw (rotation around the vertical axis)
Rz: pitch (rotation around a horizontal axis directed mediolaterally passing through the mandibular condyles of the lower jaw)
SC: Slow closing phase
SO: Slow opening phase
XROMM: X-ray Reconstruction Of Moving Morphology
